# Borders as Crossroads: The Diverging Routes of Herbal Knowledge of Romanians Living on the Romanian and Ukrainian Sides of Bukovina

**DOI:** 10.3389/fphar.2020.598390

**Published:** 2021-02-16

**Authors:** Giulia Mattalia, Nataliya Stryamets, Anya Grygorovych, Andrea Pieroni, Renata Sõukand

**Affiliations:** ^1^ Department of Environmental Sciences, Informatics and Statistics, Ca’ Foscari University of Venice, Mestre, Italy; ^2^ Institute of Environmental Science and Technology, Autonomous University of Barcelona, Cerdanyola del Vallés, Spain; ^3^ Department of biology, chemistry and bioresources, Yuriy Fedkovych Chernivtsi National University, Chernivtsi Oblast, Ukraine; ^4^ University of Gastronomic Sciences, Pollenzo, Italy; ^5^ Medical Analysis Department, Tishk International University, Erbil, Iraq

**Keywords:** Carpathians, cross-border, cross-cultural, plant-based remedies, local medicine, ethnomedicine, knowledge transfer

## Abstract

Cross-border and cross-cultural ethnomedicine are novel ways to address the evolution of local ecological knowledge. As is widely acknowledged, ethnomedicinal knowledge is not static, but evolves according to several factors, including changes in ecological availability and socioeconomic conditions, and yet the effect of the political context on medicinal knowledge remains largely underexplored. Bukovina, a small region of Eastern Europe that has been divided by a border since the 1940s and is currently part of both Romania and Ukraine, represents a unique case study in which to address the impact of political contexts on ethnomedicinal knowledge. The aim of this study was to compare plant-based medicinal uses among Romanians living on the two sides of the Romanian–Ukrainian border. In addition, we performed cross-cultural and cross-border analysis with published data on the ethnomedicine of the neighboring ethnolinguistic group of Hutsuls. We conducted 59 semistructured interviews with conveniently selected Romanians living in both Romanian and Ukrainian Bukovina. We elicited preparations for treating different ailments and disorders by naming each part of the body. We also asked about the sources of this medicinal knowledge. We documented the medicinal use of 108 plant taxa belonging to 45 families. Fifty-four taxa were common to both Romanian communities; 20 were only found among Romanians living in Romania and 34 only among Romanians living in Ukraine. However, the number of recorded uses was higher among Romanians living in Romania, revealing that they make consistent use of local medicinal plants, and Romanians living in Ukrainian Bukovina use more taxa but less consistently. Comparison with the data published in our study on neighboring Hutsuls shows that medicinal knowledge is more homogeneous among Hutsuls and Romanians living in Ukraine, yet many similar uses were found among Romanian communities across the border. We argue that the 50 years during which Ukrainian Bukovina was part of the USSR resulted in the integration of standard pan-Soviet elements as evidenced by several plant uses common among the groups living in Ukraine yet not among Hutsuls and Romanians living in Romania.

## Introduction

Local medical systems are part of a larger local ecological knowledge corpora held by local communities. According to Díaz-Reviriego et al. (2016), those medical systems are based on two elements: the availability of local resources considered “medicinal,” which generally derive from mineral products, animals, plants, or mushrooms, and ethnomedicinal knowledge, which is developed from the long-standing interaction of a community with the surrounding environment. Therefore, local medical systems are especially fostered by communities living in highly biodiverse contexts, such as the Carpathian Mountains, whose richness results from a combination of several factors, including altitude gradient, geographical position, geomorphology, and landscape heterogeneity (Mráz and Ronikier 2016).

The richness of landscapes is fostered by complex interactions developed over the centuries by local mountain communities and is an important characteristic of the Carpathian area (Cioacă and Dinu 2010; Angelstam et al., 2013; Babai et al., 2015). In addition, landscape richness is also fostered by the wealth of cultural diversity promoted by the transnational nature of the Carpathians, where borders are often rich in biocultural diversity (Liu et al., 2020) and, thus, may promote a richer corpus of local ecological knowledge (LEK) and, specifically, medicinal knowledge.

Indeed, ethnomedicinal knowledge is not static, but evolves according to several elements, such as changes in ecological availability (Júnior et al., 2013) and socioeconomic conditions (Srithi et al., 2009; Byg et al., 2010; Andriamparany et al., 2014; Menendez-Baceta et al., 2015), yet the effect of the political context on medicinal knowledge remains largely underexplored. In fact, although it has been highlighted as an important element of the context in which LEK is produced (e.g., Posey and Dutfield 1996), it has rarely been researched and is limited to folk medicinal uses in time of war (e.g., Volpato et al., 2007; Adnan et al., 2014).

A few plant-based ethnomedicinal studies have been carried out in the Carpathian Mountains, specifically among Hungarian minorities (Papp et al., 2014a; Papp et al., 2014b), Hutsuls (Sõukand and Pieroni 2016; Mattalia et al., 2020b), and Boykos (Pieroni and Sõukand 2017), as has a historical perspective on ethnomedicine at the Polish–Ukrainian border (Kozlowska et al., 2018).

In recent times, cross-border and cross-cultural studies have received increasing attention as cultural or political borders can serve as a useful variable to detect the extent to which the different political conditions that exist in two territories may contribute to shaping the use of medicinal knowledge. In the Carpathian region, the case of Bukovina is quite unique. This historical region, which for centuries was “one” territory, was split by the Soviet Union in 1940, and since 1991, it has been part of both independent Ukraine (Northern Bukovina) and Romania (Southern Bukovina).

Within this framework, the aim of this study was to compare plant-based medicinal uses among Romanians living across the Romanian–Ukrainian border and to perform cross-cultural and cross-border analysis with the ethnomedicine of neighboring Hutsuls (Mattalia et al., 2020b) to assess whether Romanians share more medicinal knowledge with Hutsuls living in the same country (Ukraine or Romania) or with Romanians living on the other side of the border.

The specific objectives were the following:

• to document and compare medicinal plant knowledge among Romanians living across the Romanian–Ukrainian border,
• to correlate the findings on medicinal plant knowledge among Romanians with a previous study on Hutsul ethnomedicine,
• to explore how the language in which medicinal plants are mentioned may contribute to the possible influence of knowledge origin among Romanians living in Ukraine, and
• to discuss whether local ethnomedicine is more similar under the same sociopolitical conditions within the same country (among Hutsuls and Romanians) or in different countries but among the same ethnolinguistic group.

## Materials and Methods

### The Study Area

**FIGURE 1:**
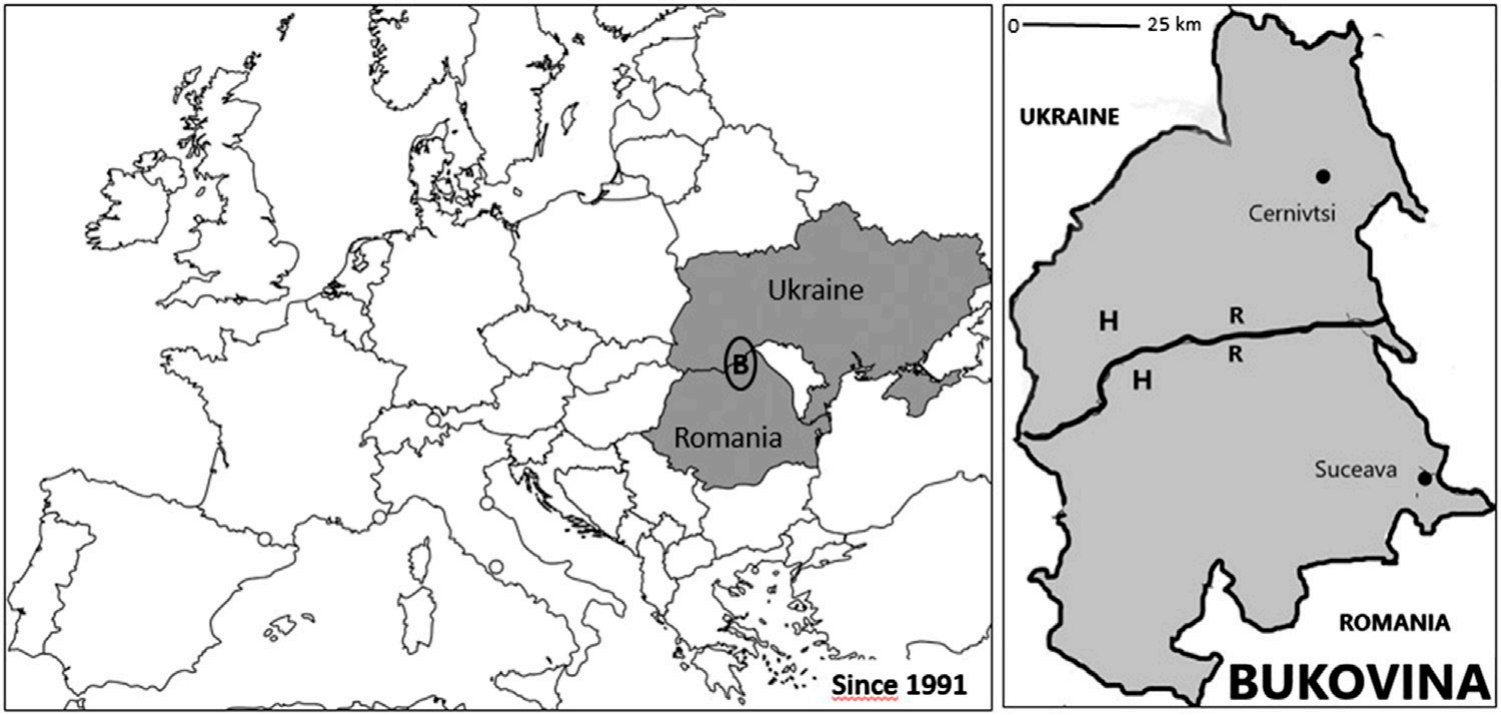
Map of the study area.

Bukovina is a historical region of Eastern Europe that partially lies in the Carpathian Mountains (Figure 1). From the second half of the 14th century to 1774, Bukovina belonged to the Principality of Moldova, after which it was occupied by the Austrian Empire until 1918 when the region became part of the Kingdom of Romania. In the 1940s, Bukovina was divided in two: The Northern part was occupied by the Soviet Union and became a territory of the Ukrainian Soviet Republic until 1991 when it became part of independent Ukraine, and Southern Bukovina has remained part of Romania.

The study area is located at the Romanian–Ukrainian border and includes one town on the Romanian side and one main town (including many parishes) on the Ukrainian side. On the European map, “B” indicates the position of Bukovina, and the map of Bukovina depicts our study area in which “R” stands for Romanians and “H” stands for Hutsuls, whose plant-based ethnomedicine is discussed in Mattalia et al. (2020b).

On both sides of the border, Romanians live in rural communities mainly devoted to family agriculture and small-scale animal husbandry. On the Ukrainian side, the effect of emigration to Western Europe is especially evident with a remittance economy starting to replace traditional activities.

## Methods

We conducted extensive fieldwork in Northern and Southern Bukovina in the summers of 2018 and 2019 (Table 1). The interviewees were conveniently selected near their homes, in their gardens, and in the street, sometimes using a snowball method. First, the study was introduced and prior oral informed consent was obtained. This study strictly followed the ethical guidelines of the International Society of Ethnobiology, and the methodology was approved by the ethics committee of Ca’ Foscari University. We conducted 59 semistructured interviews, which consisted of open-ended questions about folk medicinal uses. Current and past preparations for treating different ailments and disorders were elicited by naming each part of the body (e.g., head, ear, mouth, etc.) and asking about the mode of preparation and application. In addition, we collected background information regarding the interviewee’s age, length of time living in the area, main occupation, education, parents' native language, and religion. Finally, we inquired as to where the interviewee had learned about such medicinal uses. We attempted to ask plant by plant, but often interviewees could not remember each single use/plant and generally referred to the same sources of knowledge for every plant/use they mentioned. We conducted 17 interviews in Romanian; two in Russian; two in Ukrainian; and nine using a mixture of Russian, Ukrainian, and sometimes Romanian. Interviews primarily in Russian and Ukrainian were carried out by the second author, who is a native Ukrainian and has near-native knowledge of the Russian language. Interviews in Romanian were carried out by the first author with the help of the third author for the majority of interviews in Ukraine. In Romania, interviews were conducted with a native Romanian speaker as a facilitator.

The same methods as those employed in Mattalia et al. (2020b) were used here as both studies were carried out under the framework of the same DIGe project, which looks to assess the influence of centralization and political scenarios on the use of wild plants for medicinal purposes. In this study, 30 interviews were conducted in each area (one interview in Southern Bukovina was discarded as the interviewee was selling medicinal products made using only knowledge derived from books).

**TABLE 1:** Details of the study area.

Group	Romanians living in Romania (RR)	Romanians living in Ukraine (UR)
Number of interviews	29	30
Main municipality	Straja (Suceava region)	Krashnoilsk (Storozhenets region)
Languages of the interview	Romanian	Romanian, Russian, Ukrainian
When gathered	Summer 2019	Summers 2018 and 2019
Landscape	Hilly (400–500 m a.s.l.), mainly covered by small fields (e.g., corn), meadows and forest	Plain (200–300 m a.s.l.), mainly meadows

Whenever possible, we collected herbaceous wild voucher specimens with the help of our interviewees. The Ukrainian voucher specimens are stored in the “Roztochya” Nature Reserve (Ukraine) bearing codes NB001–NB259, and the Romanian specimens are stored in the Herbarium of Ca’ Foscari University of Venice (Italy) bearing codes SB001–SB096. Voucher specimens were identified using the The Plant List, (2013) and “Flora Europaea” (Tutin et al., 1964). Plant families were classified according to Stevens, (2001) and onward.

The responses were coded in detailed use reports (DURs) using emic categories and entered into an Excel spreadsheet for comparison. Each plant-based DUR contained interview code, language of the interview, Latin name of the plant species, local name (and its transliteration according to https://slovnyk.ua/translit.php for Ukrainian plant names and https://www.calc.ru/transliteratsyya.html for Russian plant names), language of the plant name, plant part used, preparation method, emic purpose of use, and related etic system following the ICD-11 (World Health Organization, 2019).

To perform the cross-cultural and cross-border comparison, we calculate the Jaccard index (JI) as follows: JI = (C/(A + B-C)) × 100, where C is the number of uses common to A and B, A is the number of uses in sample A, and B the number of uses of sample B (González-Tejero et al., 2008).

To calculate the proportion of each knowledge transmission strategy, we assigned a total of 1 point to each interviewee. For instance, if the interviewee reported only one source of knowledge (e.g., grandparents), we assigned a value of 1; for two sources (e.g., books and parents), we assigned 0.5 to each; three sources, 0.33 to each, etc. Then, we summed these values according to the emic categories of knowledge sources mentioned by the interviewees (folk, books, parents, etc.) on both sides of the border.

On the Ukrainian side, Romanians often spoke a mixture of languages, including Romanian, Russian, and Ukrainian. To perform the linguistic analysis, we considered only the 17 interviews conducted in Romanian. We organized each plant name according to the language in which it was mentioned. We considered 5 categories: Romanian, non-Romanian (Ukrainian or Russian), international (when a plant has very similar names in the three languages), multilanguage (when the interviewee provided the plant name in two or more languages), and dialect (when the plant name was not reported among Romanians living in Romania and a) was not included or b) was included as a dialect name in the Romanian dictionary DEX). The linguistic analysis was not performed among Romanians living in Romania as they were monolingual (Romanian). The average age of the interviewees was 60 years old in Ukraine and 63 years old in Romania. Gender distribution in both areas was 80% female and 20% male, and all interviewees were born in the Bukovina region. In both areas, the interviewees were Orthodox Christian except for two people who were Baptist. Most of the Romanian interviewees in Southern Bukovina were retired (53%) although 37% were employed outside the home, 6% worked in small-scale family farming, one person was on parental leave and one person unemployed. Only two interviewees had higher education although half of the interviewees had primary education and 12 people had secondary education. In Northern Bukovina, 45% of interviewees had primary education, 37% secondary education, 8% basic education, 5% specialist education, and 5% higher education. As in the Romanian part of Bukovina, most of the interviewees in Ukraine were retired (60%), 20% were employed outside the home, and 20% worked in small-scale family farming.

## Results

Cross-border comparison of medicinal plants used among Romanians living across the Bukovinian border.

**FIGURE 2:**
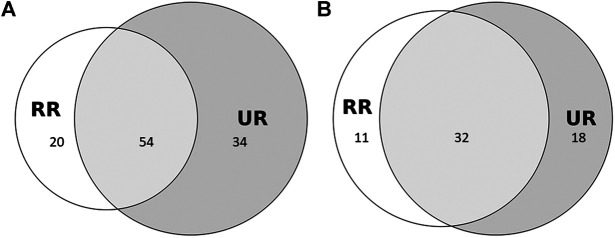
Proportional Venn diagram of taxa used by Romanians living in Romania (RR) and in Ukraine (UR). **(A)** considers all taxa, and **(B)** includes only taxa mentioned by at least 3 people. JI A = 50; JI B = 52.

We recorded the medicinal use of 108 plant taxa belonging to 45 families (Table 2). Fifty-four taxa were common to both Romanian communities, 20 were found only among Romanians living in Romania, and 34 only among Romanians living in Ukraine, corresponding to a JI of 50 (Figure 2). When we considered only those plant taxa mentioned by at least 3 interviewees (about 10% of the interviews), we observed a JI of 52 with 61 taxa in total, of which half (32) were common across the border, 11 were found only among Romanians in Romania, and 18 only among Romanians in Ukraine. Thus, 44% of the taxa were mentioned by only one or two people in each community.

**TABLE 2:** Recorded medicinal plants among Romanians living in Romania (RR) and in Ukraine (UR). Local names are in Romanian except for § (plant named in Ukrainian), @ (plant named in Russian), §@ (plant named using a term common to both Ukrainian and Russian), * (plant named with a mixture of two languages), and (plant named in the local dialect).

Latin name	Local names	Part used	Preparation	Use	System ICD-11	RR	UR
*Abies alba* Mill. (Pinaceae)	Brad	Twigs	Tea	Cold	Respiratory	2	0
			Syrup/Tea	Good for the lungs		4	0
			Syrup	Cough		6	0
				Sore throat; good for the throat		4	0
				Respiratory ways		3	0
*Achillea millefolium* L. (Asteraceae)	Coada şoricelului; Coada şoarecului; тисячелітник @	Aerial parts	Tea	Good for the stomach	Digestive	15	3
SB011	(Tysiachelitnyk @)			Good for the intestine		0	2
SB050				Diarrhea		0	1
SB074				Stomach pain		0	2
NB060				Pancreatitis		0	1
NB117				Abdominal pain		0	1
				Good for the liver		7	0
				Good for the abdomen		2	0
				Nausea		2	0
				Good for bile		1	0
				“Waking up the female side”	Genitourinary	0	1
				Women’s problems		3	3
				Good for the kidneys		3	1
				Bladder problems		0	2
				Genital problems		1	0
				Good for the urinary tract		1	0
				Good for the heart	Cardiovascular	1	0
				Intestinal worms	Certain infectious diseases	2	0
				Parasites		2	0
				Pinworm		2	0
				Panacea	General health	7	1
				Relaxing	Nervous	1	0
*Acorus calamus* L. (Acoraceae)	Zmăoaică	Roots	Tea	Good for the stomach	Digestive	0	2
			Infused in alcohol	Good for the stomach		0	3
				Abdominal pain		0	2
				Good for kidneys	Genitourinary	0	1
*Aesculus hippocastanum* L. (Sapindaceae)	Caştan; каштан § (Kashtan §)	Flowers	Infused in alcohol and locally applied	Warts	Certain infectious diseases	0	2
				Good for the joints	Musculoskeletal	0	6
SB057				Good for the skin		0	2
NB067		Fruits		Good for the joints		0	2
*Allium cepa* L. (Amaryllidaceae)	Ceapă; цибуля § (Tsybulia §)	Bulbs	Fresh	Lowering blood pressure	Circulatory	2	0
				Relaxing	Nervous	2	0
			Tea/Fresh	Cough	Respiratory	0	2
			Fomentation	Healthy	General health	0	1
		Whole plant	Any preparation	Thrombus	Circulatory	0	1
*Allium sativum* L. (Amaryllidaceae)	Usturoi; чеснок@ (Chesnok@)	Bulbs	Fresh	Good for the heart	Cardiovascular	3	0
				Blood thinning	Circulatory	2	0
				Cold	Respiratory	0	2
*Allium ursinum* L. (Amaryllidaceae)	Usturoi de padure; Leurdă; Usturoiul ursului	Leaves	Fresh in salad	Detox	General health	2	0
				Good for immunity	Immune	2	0
*Alnus* spp. (Betulaceae)	Arin	Twigs	Tea	Diarrhea	Digestive	0	1
*Aloe* spp. (Xanthorrhoeaceae)	Aloe; алоє § (aloie §)	Leaves	Infused in alcohol	Stomach ache	Digestive	0	1
			Ointment with fat, locally applied	Hemorrhoids		0	1
			Fresh, locally applied	Warts	Certain infectious diseases	1	0
				Good for the skin	Integumentary	0	2
*Althaea officinalis* L. (Malvaceae)	Nalbă mare	Flowers	Tea	Good for the colon	Digestive	1	0
*Anethum graveolens* L. (Apiaceae)	Mărar; криΠ § (Kryp §)	Seeds	Tea	Pain relief	General health	0	2
SB032				Good for the stomach	Digestive	0	2
				Children’s abdominal pain and intestinal gas		2	0
			Aerial parts	Good for the stomach		0	1
				Lowering blood pressure	Circulatory	0	2
*Arctium lappa* L. (Asteraceae)	Brusture; лоΠуχ § (Lopukh §)	Roots	Fresh	Panacea	General health	1	0
SB052			Infused in alcohol	Rheumatism	Musculoskeletal	2	0
SB091				Cancer	Neoplasm	2	0
			Tea	Wounds	Integumentary	0	1
		Leaves	Tea	Good for the stomach	Digestive	2	0
				Good for bile		2	0
			Gently pressed and locally applied	Varices	Circulatory	1	0
				Heel pain		0	1
				Joint pain	Musculoskeletal	1	3
				Headache	Nervous	2	3
				Foot pain		2	0
				Fever	General health	0	1
				Warts	Certain infectious diseases	0	2
				Heel cracking	Integumentary	0	1
		Aerial parts	Boiled	Good for the hair	Integumentary	0	5
*Armoracia rusticana* P.Gaertn., B.Mey. and Scherb. (Brassicaceae)	Hrean	Leaves	Boiled	Blood cleansing	Hematopoietic	0	1
SB031		Roots	Locally applied	Joint pain	Musculoskeletal	0	1
NB028							
*Arnica montana* L. (Asteraceae)	Arnica		Infused in alcohol	Good for the stomach		2	0
		Roots		Ulcer	Digestive	2	0
		Flowers		Promoting cicatrization	Integumentary	0	1
*Artemisia absinthium* L. (Asteraceae)	Pelin	Aerial parts	Bath	Good for the feet	General health	1	0
SB005				Women’s problems	Genitourinary	1	0
			Tea	Detox	General health	1	0
				Blood cleansing	Hematopoietic	1	0
				Good for bile	Digestive	1	0
				Good for the stomach		4	0
				Good for the liver		2	0
				Hepatitis		2	0
			Fresh	Worms (in children)	Certain infectious diseases	0	1
			Infused in alcohol	Organism cleansing	General health	0	2
*Artemisia dracunculus* L. (Asteraceae)	Tarhon	Aerial parts	Tea	Panacea	General health	0	2
SB015							
SB029							
*Asplenium scolopendrium* L. (Aspleniaceae)	Limba cerbului	Aerial parts	Tea	Good for the liver	Digestive	1	0
				Good for the stomach		1	0
				Good for the heart	Cardiovascular	1	0
				Vascular diseases		1	0
				Good for the blood	Hematopoietic	1	0
*Avena sativa* L. (Poaceae)	Овсянка @ (ovsianka)	Seeds	Tea	Podagral	Musculoskeletal	0	1
				Diarrhea	Digestive	0	1
				Constipation		0	1
*Beta vulgaris* L. (Amaranthaceae)	Sfeclă roşie	Tubers	Fresh	Cough	Respiratory	1	0
SB026				Anemia	Hematopoietic	1	0
			Juice	Anemia	Hematopoietic	1	0
				Cough	Respiratory	1	0
				Cancer	Neoplasm	0	2
*Betula pendula* Roth. (Betulaceae)	Mesteacăn; береза§ (bereza)	Buds and small leaves	Tea	Good for vessels	Circulatory	0	2
SB087				Good for the prostate	Genitourinary	0	2
NB155		Sap	Fresh	Good for the gallbladder	Digestive	1	0
NB040				Good for the kidneys	Genitourinary	1	0
				Panacea	General health	3	0
				Organism cleansing		0	2
*Bidens tripartita* L. (Asteraceae)	Turiţă; череда § (chereda §)	Aerial parts	Bath	Good for the skin	Integumentary	0	4
NB090				Skin cleansing		0	4
			Tea	Allergies	Immune	0	2
				Diabetes	Endocrine	0	1
				Blood cleansing	Hematopoietic	0	1
*Brassica oleracea* L. (Brassicaceae)	Varză (verde); Curechi; каΠуста § (kapusta §)	Leaves	Gently pressed and locally applied	Joint pain	Musculoskeletal	6	6
				Gout		0	2
*Calendula officinalis* L. (Asteraceae)	Gălbenele; нагідки § (Nahidky §)	Flowers	Tea	Good for bile	Digestive	2	0
NB170				Good for the liver		10	1
				Sore throat	Respiratory	0	1
				Headache	Nervous	1	0
				Bone pain	Musculoskeletal	1	0
				Genital problems	Genitourinary	1	0
				Disinfecting	General health	3	0
				Good for the heart	Cardiovascular	2	0
			Bath	Good for the colon	Digestive	1	0
				Women’s problems	Genitourinary	0	2
			Ointment	Good for the skin/Dry skin	Integumentary	9	0
				Dried heels		1	0
				Pimples		1	0
				Burns		1	0
				Warts	Certain infectious diseases	2	0
				Cold	Respiratory	1	0
				Varices	Circulatory	1	0
				Foot/Hand/Leg/Joint pain	Musculoskeletal	3	1
				Dislocation	Injury	1	0
				Sores	General health	1	0
				Panacea		0	1
				Mastitis	Genitourinary	0	1
			Tampon	Cervix	Genitourinary	0	1
*Capsella bursa-pastoris* (L.) Medik. (Brassicaceae)	Traista ciobanului; Πастуша сумка § (Pastusha sumka §)	Aerial parts	Boiled	Incontinence	Genitourinary	0	1
SB012			Tea	Panacea	General health	1	0
NB218				Women’s problems	Genitourinary	1	0
*Capsicum annuum* L. (Solanaceae)	Chiparuşcă &	Fruits	Any preparation	Thrombus	Circulatory	0	2
*Carpinus betulus* L. (Betulaceae)	⌈раб § (Hrab §)	Wood	Burned for soap	Body cleansing	General health	0	2
*Carum carvi* L. (Apiaceae)	Săcărică; Secărica; Chimion; кмин § (kmyn §)	Aerial parts (including seeds)	Infused in alcohol	Healthy	General health	1	0
SB007			Tea	Diarrhea	Digestive	10	0
NB037				Good for the stomach/Stomach pain		12	12
				Stomach closing		4	0
				Good for the liver		1	0
				Abdominal pain		2	0
				After giving birth	Pregnancy	1	0
				Panacea	General health	1	0
				Organism cleansing		1	0
				Cough	Respiratory	0	2
*Chelidonium majus* L. (Papaveraceae)	Rostopasca; чистотел @ (Chistotel @)	Aerial parts	Tea	Good for the liver	Digestive	9	2
SB003				Good for the prostate	Genitourinary	1	0
NB154				As an antibiotic	Immune	1	0
				Joint pain	Musculoskeletal	1	0
				Sore throat	Respiratory	1	0
				Ulcer	Digestive	0	2
			Ointment	Hemorrhoids	Digestive	0	1
			Infused in alcohol	Good for bile	Digestive	2	0
				Cancer	Neoplasm	0	1
		Sap	Locally applied	Warts	Certain infectious diseases	4	1
				Cuts	Integumentary	1	0
				Good for the eyes	Visual	0	1
		Roots	Tea	Women when weak	General health	0	2
*Cichorium intybus* L. (Asteraceae)	Cicoarea	Flowers	Tea	Good for the abdomen	Digestive	2	0
SB046				Good for the intestine		2	0
				Constipation		2	0
*Crataegus monogyna* Jacq. (Rosaceae)	Păducel; боярышник@; бояришник @ глід§; Malaieş (boiaryshnyk @; boiaryshnik@, hlid)	Twigs	Tea	Blood pressure normalization	Circulatory	6	3
SB064		Flowers and fruits		Good for the heart	Cardiovascular	12	5
NB234		Flowers		High blood pressure	Circulatory	0	4
				Good for the liver	Digestive	0	2
*Cucumis melo* L. (Cucurbitaceae)	Pepene	Fruits	Locally applied	Good for the skin	Integumentary	0	1
*Cucumis sativus* L. (Cucurbitaceae)	Огірок § (Ohirok §)	Stems	Tea	Calmant (for children)	Nervous	0	2
*Cucurbita pepo* L. (Cucurbitaceae)	гарбуз § (Harbuz §)	Seeds	Fresh	Good for the stomach	Digestive	0	2
			Tea	Stimulate appetite	General health	0	2
				Prostatitis	Genitourinary	0	2
*Daucus carota* L. (Apiaceae)	Morcov	Roots	Fresh	Improve vision	Visual	3	0
			Juice	Blood cleansing	Hematopoietic	0	1
				Cancer	Neoplasm	0	2
*Dipsacus pilosus* L. (Caprifoliaceae)	Scaius	Aerial parts	Locally applied	Joint pain	Musculoskeletal	0	2
*Elaeagnus rhamnoides* (L.) A. Nelson (Elaeagnaceae)	Cătina	Fruit	Syrup	Immune system	Immune	4	0
			Tea	Cough	Respiratory	2	0
				Blood pressure normalization	Circulatory	1	0
			Dried	Panacea	General health	2	0
*Equisetum arvense* L. (Equisetaceae)	Barba ursului; Coada calului; xвощ Πольовий § (Khvoshch polovyi §)	Aerial parts	Tea	Blood cleansing	Hematopoietic	1	0
SB020				Panacea	General health	4	0
NB093				Good for the kidneys	Genitourinary	5	4
				Water eliminating		2	0
				Genital problems		1	0
				Good for the prostate		1	0
				Good for the bladder		1	4
			Bath	Food and hand pain	Musculoskeletal	0	2
*Fragaria vesca* L. (Rosaceae)	Fragi; Frăguţ; земляніка@; ягоди§ (Zemlianika @; yahody §)	Whole plant	Tea	Panacea	General health	1	0
SB094		Fruits	Syrup/Tea	Healthy	General health	0	2
NB071			Tea/Fresh	Fever	General health	0	2
			Fresh	Vitamin provider	Endocrine	0	2
		Fruits/Aerial parts	Tea/Fresh	Good for the heart	Cardiovascular	0	1
*Galium verum* L. (Rubiaceae)	Sânzâiene	Aerial parts	Tea	Good for the thyroid	Endocrine	0	1
SB093							
NB150							
*Ginkgo biloba* L. (Ginkgoaceae)	гінго білоба § (Hinho biloba §)	Aerial parts	Tea	Good for the brain	Nervous	0	1
*Helianthus annuus* L. (Asteraceae)	Floarea-soarelui	Seeds	Oil, locally applied	Good for the ear	Auditory	2	0
*Helianthus tuberosus* L. (Asteraceae)	ТоΠинамбур § (Topynambur §)	Tuber	Tea	Joint pain	Musculoskeletal	0	2
				Leg pain		0	2
*Helichrysum maracandicum* Popov (Asteraceae)	Безсмертник @ (Beszmertnik)	Aerial parts	Tea	Good for the liver	Digestive	0	2
				Good for the stomach		0	2
*Hordeum* spp. (Poaceae)	Orz	Seeds	Locally applied	Back pain	Musculoskeletal	0	1
*Humulus lupulus* L. (Cannabaceae)	Hamei	Flowers	Tea	Blood pressure normalization	Circulatory	1	0
SB081							
NB163							
*Hypericum* spp. (Hypericaceae)	Pojarniţa; Sunătoare; зверобой@; звіробій § (Zveroboi @; zvirobii §)	Aerial parts	Tea	Good for the stomach/Stomachache	Digestive	13	16
SB092				Diarrhea		0	3
SB068				Gastritis		0	2
NB148				Pancreatitis		0	1
				Good for the abdomen/Abdominal pain		0	4
				Indigestion		2	0
				Good for the liver		4	0
				Panacea	General health	5	1
				Healthy		0	2
				Organism cleansing		1	0
				Diabetes	Endocrine	2	0
				Relaxing	Nervous	4	3
				Headache		3	0
				Women’s problems	Genitourinary	3	0
				Sore throat	Respiratory	1	0
				Good for the heart	Cardiovascular	1	0
*Juglans regia* L. (Juglandaceae)	Nuc; Γоріx § (gorih§)	Leaves	Tea	Good for the hair	Integumentary	6	0
SB051		Husk		Good for the hair	Integumentary	1	0
NB153		Fruits		Cough	Respiratory	0	1
		Unripe fruits	Infused in alcohol	Diarrhea	Digestive	0	1
			Syrup	Tongue cuts (in children)		0	1
*Lamium album* L. (Lamiaceae)	Urzică cu floare albă; Urzică înflorită; Urzică moartă; мертвая урдзика (Mertvaia urdzyka)*	Aerial parts	Tea	Women when weak	General health	0	2
SB025			Aerial parts	Women’s problems	Genitourinary	1	0
*Lavandula* spp. (Asteraceae)	Lavanda	Flowers	Mixed tea/Bath	Relaxing	Nervous	1	1
*Leonurus cardiaca* L. (Lamiaceae)	Talpa gâştei	Aerial parts	Tea	Good for the heart	Cardiovascular	0	1
				Relaxing	Nervous	0	1
*Levisticum officinale* W.D.J. Koch (Apiaceae)	Leuştean	Aerial parts	Tea	Weight-loss	Endocrine	2	0
SB030			Infused in white wine	Detox	General health	2	0
*Lilium candidum* L. (Liliaceae)	Lilia albă*; Crin; Crin alb; лилия@; лілія§ (Lilia§@)	Flowers	Infused in alcohol	Burns	Integumentary	0	1
SB049				Wounds		0	1
				Good for the skin		0	5
				Cuts		4	0
				Warts	Certain infectious diseases	9	3
				Joint pain	Musculoskeletal	2	2
				Disinfecting	General health	2	0
*Linum usitatissimum* L. (Linaceae)	Лен § (Len §)	Seeds	Tea	Podagra	Musculoskeletal	0	1
			Boiled	Abscesses	Certain infectious diseases	0	2
			Tea	Gastritis	Digestive	0	2
			Boiled and locally applied	Joint pain	Musculoskeletal	0	1
				Wounds	Integumentary	0	2
*Lycopodium clavatum* L. (Lycopodiaceae)	Pedicuţa	Aerial parts	Tea	Joint pain	Musculoskeletal	2	0
SB053				Good for the liver	Digestive	3	0
SB054				Jaundice		2	0
*Malus domestica* L. (Rosaceae)	Mere	Fruits	Vinegar	Promoting digestion	Digestive	2	0
SB038			Juice	Gall stones		0	1
				Blood cleansing	Hematopoietic	0	1
			Uzvar	Healthy	General health	0	1
*Malva* spp. (Malvaceae)	Nalbă	Aerial parts (including flowers)	Tea	Cough	General health	0	2
SB024				Bronchitis	Respiratory	0	1
NB199				Good for the throat		0	2
*Matricaria chamomilla* L. (Asteraceae)	Muşeţel; Romaniţă; ромашка § (Romashka §)	Aerial parts	Tea	Constipation	Digestive	3	0
SB002				Good for the intestine		2	1
SB019				Good for the teeth		2	2
NB171				Colic		1	0
				Good for the stomach		1	7
				Diarrhea		0	2
				Gastritis		0	2
				Good for the abdomen/Abdominal pain		0	2
				Tooth removal		0	2
				Stomach cleansing		0	2
				Jaundice		0	1
				Body cleansing (children)	Integumentary	3	5
				Wounds		0	2
				Cold	Respiratory	1	0
				Cough		1	1
				Good for the throat/Sore throat		3	1
				Disinfecting	General health	7	0
				Warming up		0	1
				Panacea		2	5
				Good for children		0	1
				Pain relief (children)		0	2
				Fever		0	8
				Relaxing	Nervous	2	0
				Good for the prostate	Genitourinary	1	0
				After giving birth	Pregnancy	2	0
				Flu	Certain infectious diseases	0	1
				As an antibiotic	Immune	0	1
			Gargling with honey	Sore throat	Respiratory	2	0
			Bath	Women’s problems	Genitourinary	3	0
				Hemorrhoids	Digestive	0	1
			Ointment	Good for the skin	Integumentary	2	0
			Locally applied	Eye cleansing	Visual	4	1
				Conjunctivitis		2	0
				Warts	Certain infectious diseases	2	0
				Dried heels	Integumentary	1	0
*Melissa officinalis* L*.* (Lamiaceae)	Melisa	Aerial parts	Tea	Good for the heart	Cardiovascular	0	2
SB095				Relaxing	Nervous	0	1
*Mentha* sp. (Lamiaceae)	Izma; Menta calului; Mintă; Menta chiparata нінта&; минти&; (Ninta&; mynty&)	Leaves	Tea	Good for the heart	Cardiovascular	0	2
SB014				Relaxing	Nervous	4	8
SB016				Headache		1	0
SB034				Panacea	General health	3	0
SB096				Diarrhea	Digestive	2	0
NB172				Good for the stomach		5	0
NB025				Good for the abdomen/Abdominal pain		2	0
				Good for the liver		3	0
				After giving birth	Pregnancy	1	0
				Flu	Certain infectious diseases	0	1
			Infused in alcohol	Mosquito bites	General health	0	1
				Cancer	Neoplasm	0	1
*Ocimum basilicum* L. (Lamiaceae)	Busuioc	Aerial parts	Bath	Ringworm	Certain infectious diseases	2	0
			Tea	Fever	General health	0	1
			Tea	Good for children		1	0
			Tea	Depression	Mental	2	0
			Tea	Headache	Nervous	2	0
*Origanum vulgare* L. (Lamiaceae)	Sovârf; şovarv; материнка§ (materynka §)	Aerial parts	Tea	Flu	Certain infectious diseases	0	1
SB036				Women’s problems	Genitourinary	0	4
NB099				Breathing	Respiratory	0	1
				Bronchitis		0	1
				Good for the lungs		0	1
				Panacea (99 diseases/17 diseases)	General health	2	2
				Good for organism		0	1
				Good for the stomach	Digestive	1	1
				Headache	Nervous	2	0
*Petroselinum crispum* (Mill.) Fuss (Apiaceae)	Pătrunjel; Πетрушка § (Petrushka §)	Aerial parts	Tea	Good for the teeth	Digestive	1	0
SB033				Detox	General health	2	0
				Wounds	Integumentary	0	1
		Roots		After giving birth	Pregnancy	1	0
				Women’s problems	Genitourinary	0	2
				Good for the kidneys		0	3
				Prostatitis		0	2
				Weight-loss	Endocrine	0	2
*Phaseolus vulgaris* L. (Leguminosae)	Fasole	Pod exocarp	Tea	Weight-loss	Endocrine	1	0
*Picea abies* (L.) H. Karst. Possibly including *Abies alba* Mill (Pinaceae)	Molid; Xвоя § (Khvoia § needle)	Twigs	Syrup	Good for the lungs	Respiratory	5	0
				Fever (children)	General health	2	0
SB008				Sore throat	Respiratory	5	0
SB021				Cold	Respiratory	1	0
NB043			Tea/Syrup	Cough	Respiratory	8	0
				Panacea	General health	2	0
			Tea	Good for the liver	Digestive	1	0
			Bath	Hemorrhoids	Digestive	0	1
				Hand and Foot pain	Musculoskeletal	0	1
		Needles	Bath	Joint pain	Musculoskeletal	0	2
				Relaxing	Nervous	0	2
*Plantago lanceolata* L. (Plantaginaceae)	Pătlagină îngustă; Limba soacrei; Minciuna	Leaves	Tea	Cough	Respiratory	1	0
SB037		Roots	Tea	Good for the lungs	Respiratory	2	0
*Plantago major* L. (Plantaginaceae)	Pătlagină (lată)	Leaves	Syrup	Panacea	General health	2	0
SB066				Good for the throat	Respiratory	1	0
NB161				Good for the respiratory ways		2	0
			Syrup/Tea	Cough	Respiratory	4	3
			Tea	Good for the stomach	Digestive	0	1
				Gastric diseases		0	1
				Bronchitis	Respiratory	0	1
			Locally applied	Wounds	Integumentary	0	4
				Cuts	Integumentary	1	0
				Hand and Foot pain	Musculoskeletal	0	2
				Good for the skin	Integumentary	0	4
				Warts	Certain infectious diseases	7	1
				Skin disease (roza)	Integumentary	0	1
				Disinfecting	General health	0	1
*Potentilla anserina* L. (Rosaceae)	Coada racului	Whole plant	Tea	Panacea	General health	1	0
*Primula veris* L. (Primulaceae)	Ciuboţica cucului; Πервоцвіт § (Pervotsvit §)	Flowers	Tea	Cough	Respiratory	1	0
				Liver problems	Digestive	0	2
		Aerial parts	Tea	Good for the abdomen	Digestive	1	0
				Good for the organism	General health	1	0
				Relaxing	Nervous	2	0
				Good for the lungs	Respiratory	2	0
*Prunus avium* (L.) L. (Rosaceae)	Cireş	Stalks	Tea	Good for the kidneys	Genitourinary	3	0
SB059				Good for the urinary tract		2	0
*Prunus cerasus* L. (Rosaceae)	Vişine; Cireş amar; вишня § (Vyshnia §)	Twigs	Tea	Flu	Certain infectious diseases	0	2
SB045				Fever	General health	0	2
				Healthy		1	0
				Cold	Respiratory	0	2
		Fruits	Infused in alcohol and sugar	Diarrhea	Digestive	0	1
				Good for the stomach	Digestive	0	1
			Infused in alcohol	Headache	Nervous	2	0
		Stalks	Tea	Jaundice	Digestive	2	0
				Good for the liver		2	0
				Good for the kidneys	Genitourinary	1	0
*Prunus domestica* L. (Rosaceae)	Prun; Perja	Seeds	Fresh	Parasites	Certain infectious diseases	1	0
				Organism cleansing	General health	1	0
		Fruits	Fresh	Constipation	Digestive	0	1
			Uzvar	General health	General health	0	2
*Pyrus communis* L. (Rosaceae)	Pere	Fruits	Uzvar	Healthy	General health	0	1
SB080							
*Quercus* spp. (Fagaceae)	Stejar; дуб § (Dub §)	Bark (and leaves)	Bath	Hemorrhoids	Digestive	0	3
SB056							
NB160							
*Raphanus sativus* L. (Brassicaceae)	Ridiche neagră; редька § (Redka §)	Roots	Boiled	Cough	Respiratory	0	1
			Raw	Good for the liver	Digestive	2	0
				Good for the kidneys	Genitourinary	2	0
				Healthy	General health	2	0
*Rheum rhaponticum* L. (Polygonaceae)	Ravint	Stalks	Syrup	Good for the liver	Digestive	2	0
				Good for ulcers		2	0
*Rhododendron myrtifolium* Schott & Kotschy (Ericaceae)	Bujor de munte	Flowers	Syrup	Asthma	Respiratory	1	0
				Bronchitis		1	0
				Cold (in children)		1	0
*Rhus typhina* L. (Anacardiaceae)	No name	Flowers	Boiled	Abdominal pain	Digestive	0	1
				Good for the stomach		0	1
*Ribes nigrum* L. (Grossulariaceae)	Смородіна §; кокци де нягри (Smorodina §; koktsy de niahry &)	Fruits	Fresh	Good for the heart	Cardiovascular	0	1
SB043				High blood pressure	Circulatory	0	3
				Blood pressure normalization		0	2
			Tea/Syrup	Healthy	General health	0	4
*Robinia pseudoacacia* L. (Leguminosae)	Salcâm	Flowers	Tea	Good for the lungs	Respiratory	2	0
SB041				Cough (children)		0	1
				Cold		1	0
				Good for the heart	Cardiovascular	3	0
				Relaxing	Nervous	6	0
				Panacea	General health	1	0
				Good for children		2	0
		Leaves	Tea	Good for the liver	Digestive	2	0
*Rosa canina* L. (Rosaceae)	Măceeş; Cacadir; свербигузка §; (Sverbyhuzka §)	Fruits	Tea	Cold	Respiratory	1	0
SB062				Cough		1	0
NB083		Aerial parts (including flowers)		Good for the heart	Cardiovascular	0	5
				Good for the kidneys	Genitourinary	0	5
		Flowers/Roots		High blood pressure	Circulatory	0	5
				Good for the liver	Digestive	0	2
*Rubus idaeus* L. (Rosaceae)	Zmeură; малина §; маліна @ (Malyna §; malina @)	Fruits	Syrup/Fresh	Healthy	General health	2	2
SB009			Juice/Fresh/Infused in alcohol	Fever	General health	0	6
SB071		Aerial parts	Tea	Improve vision	Visual	3	0
NB082				Flu	Certain infectious diseases	0	9
				Good for the stomach	Digestive	1	0
				Women’s pains	Genitourinary	1	0
				Good for women	General health	1	0
				Fever		0	5
				Panacea		0	1
				Sleep inducing		3	0
				Cold	Respiratory	0	4
				Cough		0	1
*Rubus* spp. Including *Rubus caesius* L. (Rosaceae)	Mure; ежевика @; чорниця§ (Yezhevyka @; chornytsia §)	Aerial parts	Tea	Joint pain	Musculoskeletal	1	0
SB083				Good for the liver	Digestive	2	0
NB001				Good for the stomach		2	0
NB062				Diabetes	Endocrine	2	0
				Hair loss	Integumentary	0	1
				High blood pressure	Circulatory	0	1
		Fruits	Syrup/Fresh	Healthy	General health	2	2
			Tea	Cold	Respiratory	0	2
			Fresh	Fever	General health	0	2
*Rumex acetosa* L. (Polygonaceae)	Macriş	Aerial parts	Tea	Panacea	General health	0	1
SB076			Fresh	Bronchitis	Respiratory	0	1
NB081							
*Rumex patientia* L. (Polygonaceae)	Stejie; Stevie	Roots	Tea	Diarrhea	Digestive	4	1
				Good for the stomach		0	1
			Locally applied	Joint pain	Musculoskeletal	0	2
*Salix* spp. (Salicaceae)	Răchită; Верба § (Verba §)	Wood	Burned for soap	Body cleansing	General health	0	2
SB040		Twigs	Tea	Joint pain	Musculoskeletal	0	2
SB047				Leg pain		0	2
NB073							
*Salvia officinalis* L. (Lamiaceae)	Salvia	Leaves	Tea	Good for children	General health	1	0
SB028				Menopause	Genitourinary	1	0
			Tea	Good mood	Mental	2	0
			Tea	Relaxing	Nervous	2	0
*Sambucus nigra* L. (Adoxaceae)	Soc; бузина§ (Buzyna §)	Flowers	Tea	Cough (children)	Respiratory	3	9
SB084				Improve breathing		0	2
				Good for the lungs		2	1
				Sudorific	Integumentary	2	0
				Blood pressure normalization	Circulatory	0	2
				Flu	Certain infectious diseases	0	2
				Fever	General health	0	3
			Syrup/Tea	Cold	Respiratory	5	1
				Sore throat		6	0
				Healthy	General health	3	0
*Solanum lycopersicum* L. (Solanaceae)	Roşie	Fruits	Gargling with lemon	Sore throat	Respiratory	0	1
*Solanum tuberosa* L. (Solanaceae)	Cartofi; Barabule; картошка @ (kartoshka @)	Tuber	Locally applied	Headache	Nervous	2	4
			Fomentation	Cough	Respiratory	0	1
				Women’s problems	Genitourinary	0	2
*Solidago virgaurea* L. (Asteraceae)	Splinuţă	Aerial parts	Tea with *Lamium album*	Good for the kidneys	Genitourinary	0	1
*Sorbus aucuparia* L. (Rosaceae)	рябина@; чорная рябина @ (ryabina @; chornaia ryabina @)	Fruits	Juice/Tea	High blood pressure	Circulatory	0	5
*Symphytum officinale* L. (Boraginaceae)	Tătăneasă; Iarbă tatei &; Iarbă lui tatin &; живокост§ (Zhyvokost §)	Roots	Bath	Body cleansing	General health	0	1
SB070			Ointment	Leg pain	Musculoskeletal	0	2
NB176			Rubbed fresh/poultice/infused in alcohol	Joint pain	Musculoskeletal	3	5
NB177			Locally applied	Fracture	Injury	0	1
				Heel pain	Musculoskeletal	0	1
			Infused in alcohol	Rheumatism	Musculoskeletal	2	0
				Rubdown	General health	1	0
				Pain relief		2	0
			Tea	Healthy	General health	1	0
		Whole plant	Tea	Panacea	General health	2	0
		Leaves/Roots	Locally applied	Warts	Certain infectious diseases	3	0
		Leaves	Infused in alcohol	Dislocation	Injury, poisoning or certain other consequences of external causes	2	0
		Roots and flowers	Tea	Vascular diseases	Cardiovascular	1	0
*Syringa vulgaris* L. (Oleaceae)	Liliac	Aerial parts	Tea	Cough	Respiratory	0	1
*Taraxacum campylodes* G.E.Haglund (Asteraceae)	Păpădia; Pască gaine &; Curul găinii &; кульбаба§ Одуванчики @ (kulbaba §; Oduvanchiki @)	Roots	Tea	Asthma	Respiratory	0	2
SB063		Flowers	Syrup	Good for the throat/Sore throat	Respiratory	2	0
NB084				Bronchitis		1	1
				Good for the lungs		0	1
				Asthma		1	0
				Cold		3	0
				Good for children	General health	0	1
				Panacea		1	0
				Good for the liver	Digestive	4	0
				Liver detoxing		2	0
				Good for immunity	Immune	1	0
			Fresh	Lung problems	Respiratory	1	0
			Chewing fresh	Stomach cleansing	Digestive	0	1
			Tea	Cancer	Neoplasm	0	1
		Aerial parts	Tea	Good for the liver	Digestive	2	1
		Stems	Fresh	Good for the liver	Digestive	2	0
*Thymus* spp. Including *Thymus serpyllum* L./*Thymus vulgaris* L. (Lamiaceae)	Cimbrişor; Cimbru; чебрець§ (Chebrets §)	Aerial parts	Tea	Panacea	General health	2	0
SB001				Healthy		0	2
SB090				Headache	Nervous	1	0
NB027, NB030				Good for the stomach	Digestive	2	0
				Good for the heart	Cardiovascular	1	0
				Cold	Respiratory	0	2
				Good for the lungs		0	1
				Bronchitis		0	1
			Tea/Fomentation	Cough	Respiratory	0	7
*Tilia cordata* Mill. (Malvaceae)	Tei; лиΠа § (Lypa §)	Flowers	Bath	Body cleansing	General health	0	1
SB017			Tea	Good for the heart	Cardiovascular	1	0
NB253				Cough	Respiratory	1	0
				Sore throat		1	0
				Cold		5	2
				Good for the lungs		1	0
				Panacea	General health	0	1
				Fever (children)		3	2
				Healthy		1	0
				Diabetes	Endocrine	2	0
				Cramps	Nervous	1	0
				Relaxing		14	0
				Headache		1	0
				Flu	Certain infectious diseases	0	2
*Trifolium* spp. (Leguminosae)	Trifoi roşu; Trifoi; клевер @ (Klever@)	Aerial parts	Tea	Good for the heart	Cardiovascular	0	3
SB075				Good for the organism	General health	0	2
SB077				Headache	Nervous	0	2
SB078				Gastritis	Digestive	0	2
SB072				Good for the gallbladder		0	2
NB076			Bath	Leg and Foot pain	General health	0	1
*Tussilago farfara* L. (Asteraceae)	Podbal	Leaves	Tea	Good for the stomach	Digestive	0	1
SB065			Infused in alcohol and locally applied	Good for the skin	Integumentary	0	1
SB085							
NB070							
*Urtica dioica* L. (Urticaceae)	Urzică	Aerial parts	Tea/Fresh	Blood/Vessel cleansing	Hematopoietic	9	8
SB088		Aerial parts	Tea/Soup	Good for blood circulation	Circulatory	6	0
NB026			Shampoo	Good for the hair	Integumentary	4	6
			Soup	Vitamin provider	Endocrine	0	1
			Tea	Blood changing	Hematopoietic	1	0
				Hair loss	Integumentary	0	1
				Good for bones	Musculoskeletal	0	2
				Blood pressure normalization	Circulatory	2	0
				Good for the heart	Cardiovascular	1	0
				Good for the stomach	Digestive	1	0
				Panacea	General health	0	1
				Kidney stones	Genitourinary	0	1
				Headache	Nervous	0	1
		Whole plant	Tea/Fresh	Iron provider	Endocrine	6	0
*Vaccinium myrtillus* L. (Ericaceae)	Afina; черника @ (Chernika @)	Fruits	Fresh	Improve vision	Visual	3	0
SB006			Fresh/Jam	Good for eyes		3	2
NB060			Tea/Fresh/Infused in alcohol/Syrup	Stomach pain	Digestive	2	6
			Dried/Infused in alcohol	Diarrhea		2	3
			Jam	Pancreatitis		0	1
			Infused in alcohol	Abdominal pain		0	1
		Aerial parts	Tea	Diabetes	Endocrine	3	0
				Improve vision	Visual	8	0
				Good for the eyes		2	0
				Good for stomach	Digestive	3	0
*Vaccinium vitis-idaea* L. (Ericaceae)	Merişoare; Merişor	Aerial parts	Tea	Fever	General health	1	0
SB010				Panacea		0	1
NB061				Improve vision	Visual	2	0
				Good for the eyes		2	0
				Good for the heart	Cardiovascular	2	0
				Women’s problems	Genitourinary	2	0
*Valeriana officinalis* L. (Caprifoliaceae)	Valeriană; валеріана § (valeryana §)	Roots	Infused in alcohol/Tea	Good for the heart/Heart problems	Cardiovascular	2	1
*Viburnum opulus* L. (Adoxaceae)	Calina; калина § (kalyna §)	Fruits	Tea	Cold	Respiratory	1	0
NB157				Flu	Certain infectious diseases	1	0
			Tea/Syrup/Raw	Cough (children)	Respiratory	5	5
			Tea/Fresh	Blood pressure normalization	Circulatory	1	6
			Fresh	Tuberculosis	Respiratory	0	1
*Viola* spp. Including *Viola tricolor* L. (Violaceae)	Panseluţa; Trei fraţi pătaţi	Aerial parts	Tea	Allergies	Immune	2	0
SB079							
*Viscum album* L. (Santalaceae)	Vasc	Aerial parts	Tea	Good for the heart	Cardiovascular	2	0
*Vitis vinifera* L. (Vitaceae)	Strugure	Leaves	Tea	Diabetes	Endocrine	1	0
*Zea mays* L. (Poaceae)	Porumb; кукурудза § (kukurudza §)	Fruit	Bath	Joint pain	Musculoskeletal	0	2
				Cough	Respiratory	1	0
				Good for the kidneys	Genitourinary	4	6
				Good for the urinary tract		0	1
*Zingiber officinale* Roscoe (Zingiberaceae)	Imbir	Tuber	Tea	Organism cleansing	General health	0	2

The two most important taxa among Romanians living in Ukraine were common to the two communities and included *Matricaria chamomilla* L (47 DURs in Romania and 51 in Ukraine) and *Hypericum* spp. (39 DURs in Romania and 32 in Ukraine). The two most important taxa in Romania [*Achillea millefolium* (50 DURs) and *Calendula officinalis* (42 DURs)] were more rarely used among Ukrainian Romanians. The most important families were Asteraceae (13 taxa), followed by Rosaceae (11 taxa) and Lamiaceae (8 taxa). Among Romanians living in Romania, 58% of the taxa were wild, and this value was 53% among Romanians living in Ukraine.

Regarding the number of DURs, we recorded 18% fewer DURs among Romanians living in Ukraine as they often reported using medicines from the local pharmacy or from abroad.

### Linguistic Analysis of Plant Names Mentioned by Romanians Living in Ukraine

**FIGURE 3:**
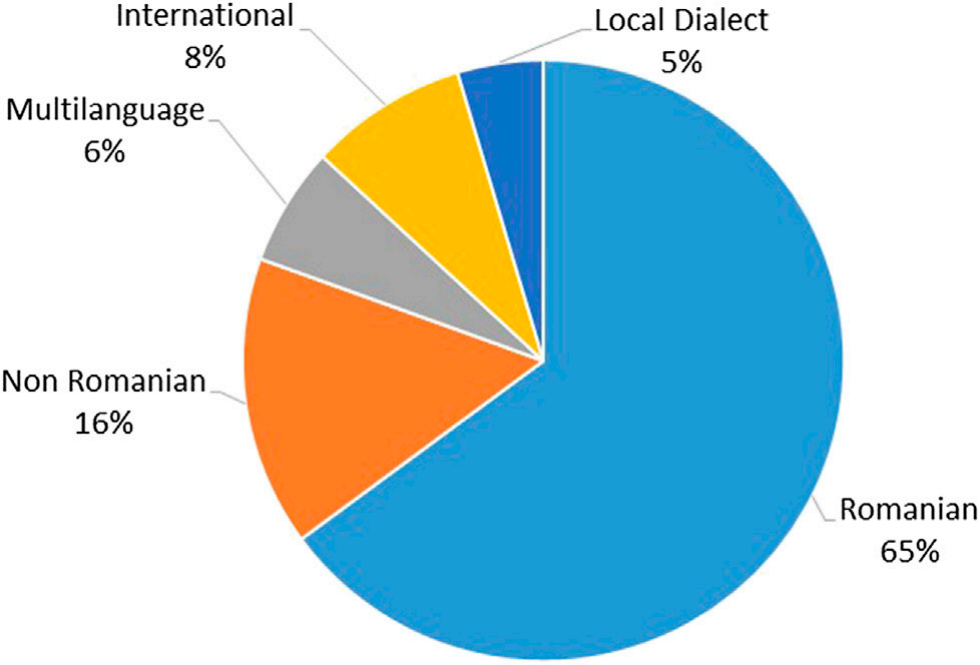
Distribution of languages used to mention medicinal plants among Romanians living in Ukraine.

Although Romanians living in Romania only speak Romanian, the linguistic analysis of plants mentioned by Romanians living in Ukraine revealed that only 65% of the plants were named in Romanian (Figure 3), whereas 16% were mentioned in Ukrainian and/or Russian, and 6% were given in multiple languages, thus providing a name in Romanian and its equivalent in one or more other languages. Eight percent were international names (e.g., *Aesculus hippocastanum*, *Viburnum opulus*, *Melissa officinalis*, *Aloe* spp.), and 5% were local dialect names, including “chiparusca” for *Capsicum annuum*, “curul găinii” for *Taraxacum officinale*, “iarba tatei” or “iarba lui tatin” for *Symphytum officinale*, “curechi” for *Brassica oleracea*, and “minciuna” for *Plantago major*. In some instances, languages were mixed within the same plant name as was the case for *Lamium album*, which was called “mertvaia urzica”—mertvaia is the Russian translation of moartă (dead) and urzică is Romanian for *Urtica dioica.* Indeed, in Romanian, “urzică moartă” is the name for *Lamium album.* A similar situation was observed for *Lilium album,* locally called “lilia alba,” which is a mixture of the Ukrainian name “lilia” (in Romanian, it would be “crin”) and the Romanian adjective “alba” (white).

### Cross-Border Comparison of Medicinal Uses Among Romanians Living Across the Bukovinian Border

**FIGURE 4:**
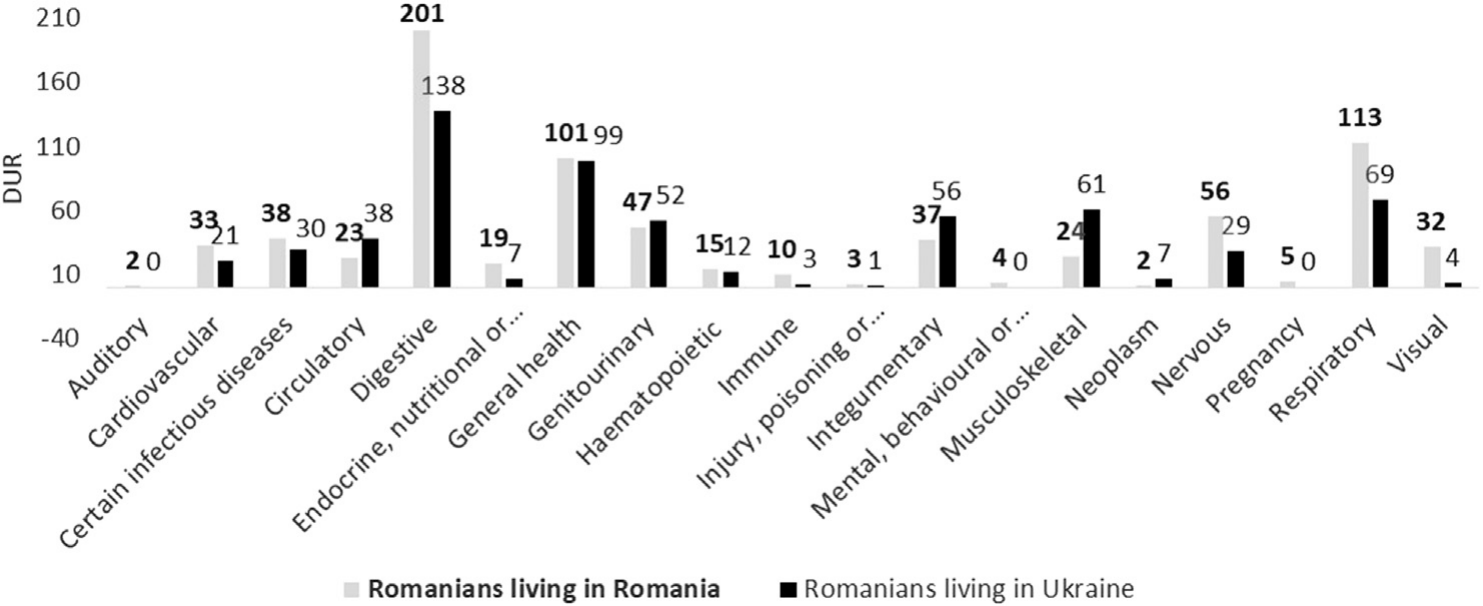
Cross-border comparison of treated systems among Romanians living in Romania (RR) and Ukraine (UR).

Among Romanians living in Romania, plant remedies were especially used for treating the digestive and respiratory systems, which correspond to emic treatments, such as “good for the stomach” and “good for the liver” or “cough” and “cold,” respectively. General health (e.g., “panacea,” “healthy,” and “good for kids”) was equally important in both communities (Figure 4). Among Ukrainian Romanians, the musculoskeletal and integumentary systems were also frequently mentioned, indicating a preference for external uses.

In both communities, tea was the most important medicinal preparation as just water and the plants themselves are needed. In Romania Bukovina, we met a 92-year-old woman who showed us the panacea tea she makes every year. She stored it in a big burlap bag along with 12 dried species, including the flowers of *Primula vulgaris*, *Primula elatior*, *Arnica montana*, *Calendula officinalis*, *Robinia pseudoacacia*, *Rosa rugosa*, and *Tilia cordata*; the stalks of *Prunus cerasus*; and the aerial parts of *Mentha* spp*.*, *Hypericum perforatum*, *Achillea millefolium*, and *Thymus serpyllum*.

In both areas of Bukovina, tea was sometimes sweetened with honey, especially for treating respiratory disorders. External uses, such as “locally applied” or “infused in alcohol and then locally applied,” as well as “raw” uses were equally important across the border although a bath preparation was considerably more important in Ukraine and syrup much more often mentioned in Romania.

Fifteen DURs related to 11 taxa were mentioned by at least 3 interviewees (10% of the sample) on both sides of the border. Both groups used *Achillea millefolium* for stomach ailments and women’s problems and *Crataegus* spp. for normalizing blood pressure and being good for the heart. Likewise, in both communities, *Equisetum* and *Zea mays* teas were used for treating the kidneys, *Lilium candidum* was infused in alcohol and locally applied to warts, and *Matricaria chamomilla* was used for body cleansing. The leaves of *Plantago major*, tea made from *Sambucus nigra* flowers, and the fruits of *Viburnum opulus* were used to treat cough among Romanians in both Romania and Ukraine. Teas made from *Mentha* spp. and *Hypericum* spp. were used as relaxants, and the roots of *Symphytum officinalis* were used to treat joint pain. *Urtica dioica* was mentioned as a shampoo. Moreover, 4 use combinations were reported identically by at least 20% of the interviewees (6 individuals) and included a cultivated species, *Brassica oleracea*, locally applied for joint pain and three wild species: *Urtica dioica* was used for blood cleansing and *Carum carvi* and *Hypericum* spp. Specifically, the last two plants were mentioned by some 40% of the interviewees for treating stomach conditions.

### Cross-Cultural Comparison of Medicinal Plant Uses with Hutsuls Living in Romanian and Ukrainian Bukovina

**FIGURE 5:**
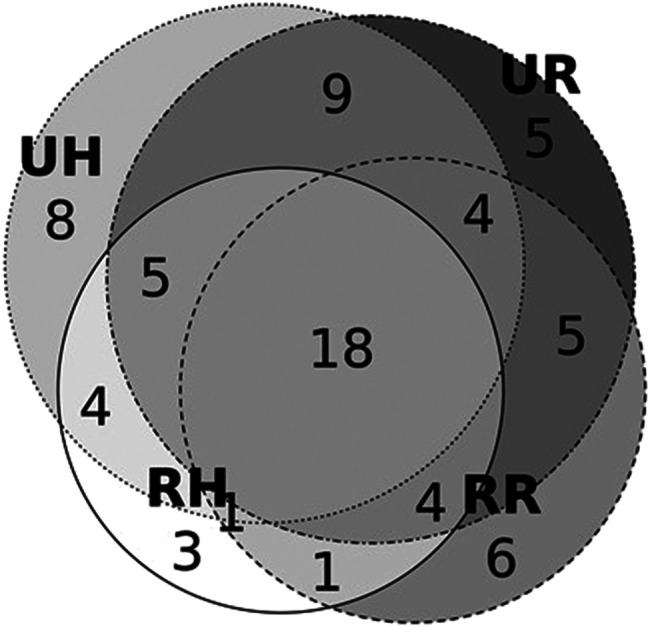
Proportional Venn diagram of taxa used by at least 3 interviewees in each community. RH = Hutsuls living in Romania, RR = Romanians living in Romania, UH = Hutsuls living in Ukraine, UR = Romanians living in Ukraine.

The comparison with data from our previous publication (Mattalia et al., 2020b) addressing a cross-border comparison among Hutsuls living in Romania and Ukraine showed that 18 taxa were common to the four communities (Figure 5). Some uses were reported by all four Bukovinian communities across the border, including *Carum carvi* and *Hypericum* spp. for stomach ailments as well as boiled *Urtica dioica* for washing hair and for cleansing the blood. Some other species growing at higher altitudes (where Hutsuls live), such as *Arnica montana* and *Vaccinium myrtillus*, were not so common among Romanians. On the contrary, the shared species are widely available and respond to common and not-so-urgent needs, such as stomach pain or hair washing.

In line with our findings among Romanians, Hutsuls living in Romania mentioned more plant-based remedies for the digestive system, and the musculoskeletal and integumentary systems were more often cited by Hutsuls living in Ukraine.

### Cross-Cultural Comparison in Romanian Bukovina

**FIGURE 6:**
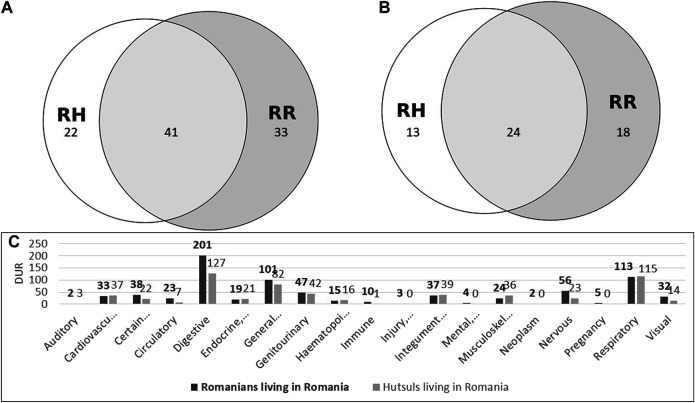
Proportional Venn diagram of taxa used by Hutsuls (RH) and Romanians (RR) living in Romania. Diagram A considers all taxa, and diagram B includes only taxa mentioned by at least 3 people. Diagram C shows the systems treated with plants among Romanians and Hutsuls living in Romania.

Hutsuls and Romanians living in Romania shared 41 plant taxa used for medicinal purposes although 22 were found only among Hutsuls and 33 only among Romanians for a total of 96 taxa (Figure 6). The JI between these two groups was 43, thus indicating less similarity than that between the two Romanian groups, i.e., those living in Romanian and Ukraine. The taxa shared exclusively among these two groups living in Romania included *Potentilla anserina*, *Prunus avium*, and *Salvia* spp. However, they were mentioned by only one or two people. Indeed, considering those plant taxa mentioned by at least 3 interviewees, only one plant (*Abies alba*) was common to only these two groups as the other 23 were also shared with the Ukrainian groups.

The analysis of the treated systems revealed that the digestive, circulatory, immune, and nervous systems were more important among Romanians, and the musculoskeletal system was more important among Hutsuls. Plant-based remedies for the cardiovascular, genitourinary, integumentary, and respiratory systems were equally reported in the two communities. In Romania, Hutsuls mentioned 22% fewer DURs than Romanians.

Regarding medicinal preparations, teas were less important among Romanians than Hutsuls (55% vs. 67%), and syrup was equally mentioned, whereas topical applications were more important among the latter group.

Of the top 5 most used plants within each community, only *Hypericum* spp. was found in common (32 DURs among Hutsuls and 39 among Romanians). The most used plant taxa among Hutsuls included *Vaccinium myrtillus*, *Urtica dioica*, *and Tilia cordata*, and among Romanians, they included *Achillea millefolium*, *Matricaria chamomilla*, and *Calendula officinalis*.

### Cross-Cultural Comparison in Ukrainian Bukovina

**FIGURE 7:**
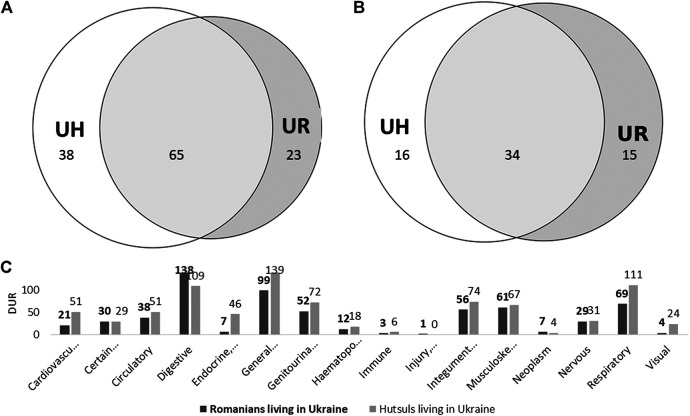
Proportional Venn diagram of taxa used by Hutsuls (UH) and Romanians (UR) living in Bukovina, Ukraine. **(A)** considers all taxa, and **(B)** includes only taxa mentioned by at least 3 people. Diagram C shows the systems treated with plants among Romanians and Hutsuls living in Ukraine.

Romanians and Hutsuls living in Ukrainian Bukovina mentioned 126 medicinal plant taxa, of which 65 were shared, 38 were found only among Hutsuls, and 23 only among Romanians (Figure 7). The related JI was 52. When considering only those taxa mentioned by at least three interviewees, the overall numbers decreased significantly to 34 common plants, 16 found only among Hutsuls, and 15 only among Romanians. However, the JI was the same at 52.

The most important treated systems were the digestive system among Romanians and the respiratory system as well as the general health category among Hutsuls. The cardiovascular, circulatory, endocrine, and visual systems were mentioned more often among Hutsuls than among Romanians. Indeed, Hutsuls reported a higher number of medicinal DURs.

Of the 5 top used plant taxa, three were found in common between the two communities (*Rubus idaeus*, *Hypericum* spp., *Urtica dioica*). The most used plant among Hutsuls was *Vaccinium myrtillus*, which was not available to Romanians, whose most used species was *Matricaria chamomilla*.

Nine taxa were mentioned as being used for medicinal purposes by at least three interviewees, all in Ukraine. They included two wild species (*Quercus* spp., *Rumex patientia*) and seven that are cultivated (*Aesculus hippocastanum*, *Aloe vera*, *Anethum graveolens*, *Linum usitatissimum*, *Malus domestica*, *Melissa officinalis*, *Ribes nigrum*).

### Knowledge Transmission, a Cross-Border and Cross-Cultural Analysis

**FIGURE 8:**
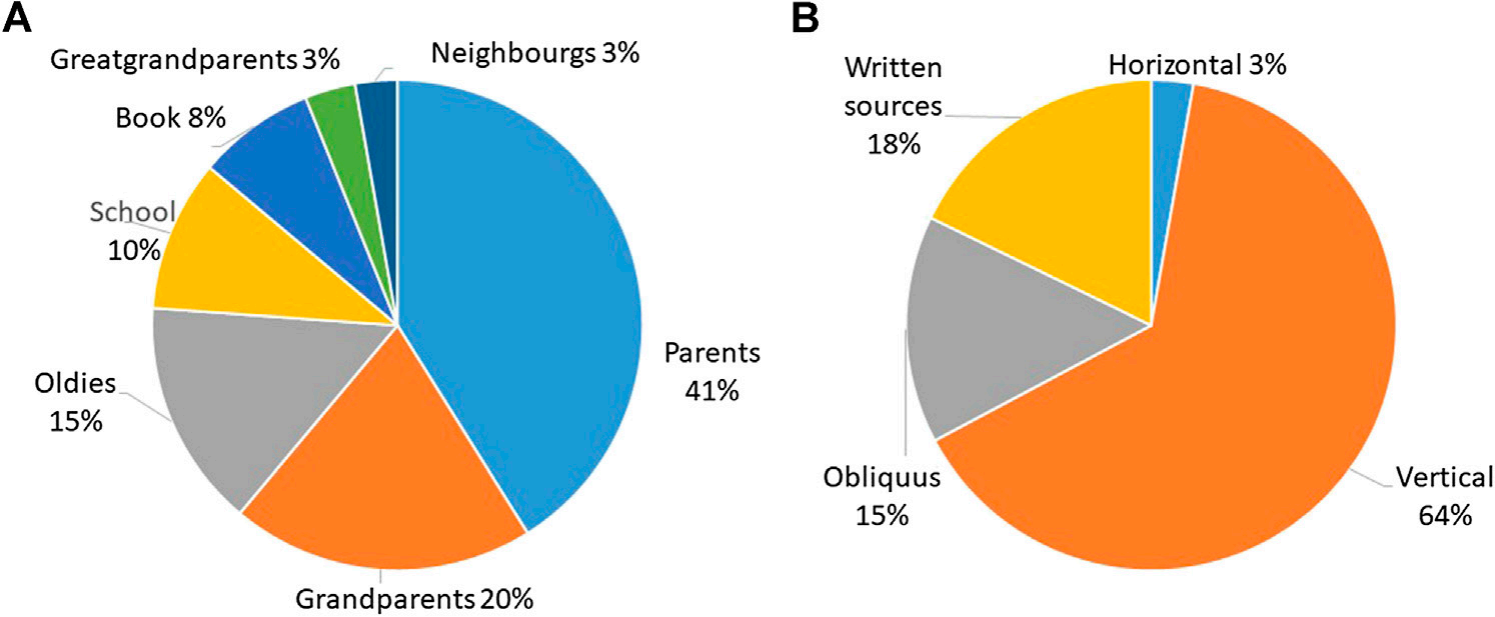
Knowledge transmission among Romanians living in Romania per emic category **(A)** and etic category adapted from Van den Boog et al. (2017) **(B)**.

We recorded seven sources of knowledge among Romanians in Romania. The most mentioned source was parents (41%), followed by grandparents (20%) and the elderly (15%), and written sources (including the category “school,” which is based on the response “biology books” provided by many interviewees) altogether accounted for 18% (Figure 8). When considering the categories proposed by Van den Boog et al. (2017), vertical transmission was predominant, followed by written sources and oblique transmission.

Comparison with the knowledge transmission strategies among Romanian Hutsuls revealed that the elderly played a major role among Hutsuls although among Romanians written sources were more important. Indeed, some Romanian interviewees living in Straja reported that they had started reading books on medicinal plants since their retirement as they have had more spare time. They already knew most of the plants, but as they have had more free time, they have deepened their knowledge and started valuing the same plants for other purposes. For instance, one person said, “I have read a lot about what they [the plants] are good for because we knew the plants from our grandparents as they used many plants for everything” (female, born 1948). Another female interviewee also reported having learned from monastery books: “I have learned from my parents, but there are a lot of books from monasteries because there they use a lot [of plants]. They [the plants] have been used for long time, but newer recipes have appeared” (female, born 1957).

**FIGURE 9:**
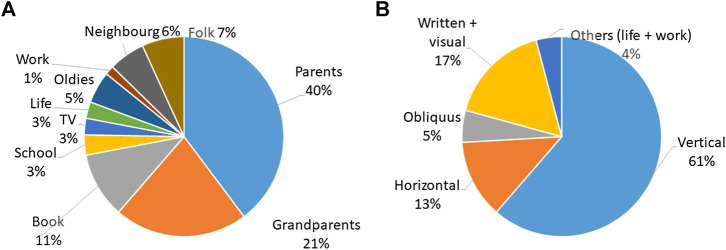
Knowledge transmission among Romanians living in Ukraine (n = 22; 8 interviews = source of knowledge unknown).

Among Romanians living in Ukraine, we recorded ten sources of knowledge (Figure 9). The most mentioned were parents, followed by grandparents (together representing vertical knowledge and 61% of the total). Horizontal knowledge (neighbors and folk) accounted for 13%, and the elderly (oblique knowledge) constituted 5%. Written and visual sources represented 17% of the sources of knowledge.

In present-day Ukraine, where culture and education are more accessible, owning a book is still a sign of social prestige, e.g., an interviewee told us, “I have learned from my parents and from school and a medicinal book. I have son who is a doctor, so I have a big book with all the plants, and I have looked through it. There are many plants we still don’t know.” During the socialist era, medicinal books were considered “scarce,” and they were very hard to buy. Also, in Ukraine, it is a point of pride to own books and to have knowledge from books as, during Soviet times, only those who had an education and books had good jobs and salaries. In addition, as a post-Soviet phenomenon, local or “grandmother” knowledge has been criticized and even satirized.

However, an important role was also assigned to television: “I have learned a lot from television. There are programs by Romanians [on Romanian TV], where doctors talk nicely [in an understandable way] about everything. Everything I told you is from books, from television, and from our life.” Although other Romanians living in Ukraine also mentioned the Romanian-language channel MEGA as a source of knowledge, the woman highlighted “from our life,” which was also mentioned by other interviewees with the expressions “from myself” or “from my job.” This concept was not mentioned among Romanians living in Romania and may underline the importance of personal strength to overcome difficult times, such as those they probably experienced during the Soviet period and the socioeconomic crises after the Soviet Union collapsed.

At the same time, among Romanians living in Ukraine, there were also some individuals who did not use books because “we read books and we forget,” which was also something mentioned among Hutsuls living in Romania. This attitude might be indicative of a rejection of that bibliophilic society in which books are a source of pride (found in the Ukrainian society), still coexisting with the perspective that “there is no time for books,” and somehow vertically transmitted knowledge is more important (found in the Romanian society). However, another factor may be the high reliance on “neighbor knowledge” or oral knowledge transmitted horizontally.

A couple of women (born in 1954 and 1960), when asked about medicinal plants, declared, “I don’t know. We bring them from Romania; in Italy, they have such a good tea mixture against cancer. My sister-in-law brings them to me from Romania, Italy, and America. Cancer is the hardest to treat.” Interestingly, remedies for cancer were mentioned by an educated couple in Romania, and it was mentioned by six interviewees in Ukraine. All the interviewees reported having learned about cancer remedies recently. At the same time, the ongoing erosion process was also mentioned by another Romanian man (born in 1935) in Ukrainian Bukovina: “I don’t know a lot. In the past, the grandmas harvested [medicinal plants], but now not too much. You go to the shop and can get everything you need.”

Comparing the ecological knowledge transmission of Hutsuls and Romanians living in Romania, we observe that the elderly were more important among the former (42%) than the latter (15%) as their societal structure is likely different—more centered at the community level than the family level—due also in part to the higher degree of remoteness of their mountain dwellings. In addition, books and schools were less important sources of knowledge among Hutsuls, representing 2% and 3%, respectively (they were 8% and 10% among Romanians).

Hutsuls and Romanians living in Ukrainian Bukovina shared a more similar way of transmitting ecological knowledge, yet the elderly were still more important among Hutsuls as were parents and grandparents. Books and TV had the same importance in the two communities.

## Discussion

### Caveats of the Study

Before discussing the results of the study, we want to mention some caveats that may affect our interpretation and were considered in the following discussion. As the interviews in Romanian were conducted with the help of facilitators, small details of the narratives could have been lost in translation. Interviews among Ukrainian Romanians were conducted partially in Romanian and partially in Ukrainian and Russian, depending on the interviewer and also the language that the interviewee preferred. However, interviewees often responded in a mixture of the three languages, which sometimes made it difficult to capture particulars of their narratives. Moreover, we conducted the linguistic analysis only among the interviews conducted in the Romanian language to avoid bias as a result of the vehicular languages of the interviews. Finally, the sample was not randomized, for which the representativeness of responses can be questioned.

### Romanian Ethnomedicine Across Borders and Cultures

The JI of plant-based ethnomedicine reveals that the closest groups are Hutsuls and Romanians living in Ukraine (JI = 52 for all taxa and for taxa mentioned by at least three interviewees) followed by Romanians on both sides of the border (JI = 52 for all taxa and JI = 50 for taxa mentioned by at least three interviewees) and Hutsuls and Romanians living in Romania have the least similarity (JI = 44 for all taxa and JI = 43 for taxa mentioned by at least three interviewees). These results indicate a stronger cohesion between the two groups living in Ukraine. We found some plant taxa shared only by the two Ukrainian groups, possibly confirming our previous hypothesis regarding the presence of some pan-Soviet influence in the ethnobotany of Ukrainian Bukovina (Mattalia et al., 2020b). Among possible pan-Soviet elements, *Aesculus hippocastanus* infused in alcohol for relieving joint and rheumatic pain was also reported in Belarus Sõukand et al. (2017a) and in Estonia (Sõukand and Kalle 2011), where an increase in use was also detected during Soviet times. Another plant common to other Eastern European countries is *Aloe* spp., which is used especially for treating the skin but also the digestive system (Sõukand et al., 2017b). *Linum usitatissimum*, well known in Estonia for its ethnoveterinary for its ethnoveterinary properties (Kalle and Kaas, 2020), is still sometimes used for the digestive system (Sõukand et al., 2017b). Finally, *Ribes nigrum* was mentioned on the Romanian side of the border but only for food purposes, and in Ukraine, it was also mentioned for treating the circulatory and cardiovascular systems, and it was reported for several other uses in Belarus (Sõukand et al., 2017b).

### What Language Can Reveal

Linguistic analysis of the plant names mentioned among Romanians living in Ukraine reveals possible links to (written) sources of knowledge in Russian and/or Ukrainian. A prime example is provided by *Arctium lappa* (“lopukh”), which was mentioned among Romanians living in Ukraine for hair care. The same use was quite popular among Ukrainian Hutsuls although, on the Romanian side, it was not mentioned at all. During Soviet times, *Arctium lappa* was actively used; there was even a state standard for collection of its roots (Spravochnik 1983). In published books, it is explained that the roots help to have “beautiful and nice hair” (Reva and Lypoveckyi 1977) and that the oil extract is used for hair care (Kharchenko et al., 1971). However, the plant described by Komendar, (1971) as good against hair loss and also skin cancer is *Arctium tomentosum*, not *Arctium lappa*, as the local name is the same. Similarly, *Avena sativa* and *Linum usitatissimum* were mentioned only among Hutsuls and Romanians living in Ukraine. *Avena sativa* was described as a source of vitamin B and good for the appetite (Olijnuk et al., 1990). *Linum usitatissimum* is described as having anti-inflammatory properties (Kharchenko et al., 1971; Spravochnik 1983) and as a remedy for gastric ulcer (Gammerman et al., 1976). Another example is provided by *Ribes nigrum*, which is referred to with the non-Romanian name “smorodina.” In Soviet books, it is described as rich in vitamins, especially vitamin C, and as antidiarrhea and diuretic remedies (Kharchenko et al., 1971; Karhut, 1976; Mamchur, 1986). Its use for treating circulatory and cardiovascular disorders was reported only in Ukraine (among both Romanians and Hutsuls). On the other side of the border, Romanians used this taxon only for food purposes, using the Romanian name “coacăze negre.” This term was also used among Romanians in Ukraine but to treat general health issues. These examples suggest that language can serve as a vector, providing a clue to the possible roots of such uses.

In addition, the linguistic analysis highlighted the use of very specific terms among Romanians living in Ukraine when compared with Romanians living in Romania. Indeed, they used such terms as “ghemoglobin” (hemoglobin), “pancratit” (pancreatitis), and “trombii” (thrombus), which were not used among Romanians living in Romania. The latter, on the contrary, used very common and basic medical terms, mainly referring to different parts of the body. Ukrainian Hutsuls mentioned several technicisms, including “cardiomagnil”.

### The Divergent Evolution of LEK Held by Romanians Living Across the Border

Another aspect revealed by the cross-border analysis involves the different knowledge transmission strategies of the different groups. In Romania (mainly Romanian) interviewees mentioned using books to supplement the knowledge they acquired from their parents, and this occurred only in people who had an above-average education and the time afforded by retirement. Basically, while enjoying more free time, they reported reading and learning about new uses for the plants they had known since childhood. An interesting comment was made by a woman who highlighted local monasteries as a source of knowledge. Indeed, in Bukovina, Orthodox monasteries have been crucial elements of the cultural landscape for many centuries (Nicu and Stoleriu, 2019). No one in Romania reported television or the Internet as a source of their knowledge. Conversely, we may describe our Ukrainian interviewees as bibliophilic as they often proudly reported having big medicinal books and using them when needed. Therefore, we can argue that the current medicinal knowledge held by Romanians living in Ukraine has an important scholarly knowledge component (*sensu* Mattalia et al., 2019) that originated in the Soviet context. Moreover, they mentioned having learned from television as well, but in our presence, they searched for answers on YouTube in the Russian language. Indeed, the era of television’s influence dates to the 1980s and 1990s when, in Soviet countries, many healers provided medical advice on various programs, and many people still believe them (Bogdanov, 2020).

Interviewees appeared proud to be able to navigate different systems, which is similarly experienced in other spheres, such as linguistics. Indeed, they declared not being able to speak any language properly as they do not speak “român curat,” or correct Romanian (literally: clean Romanian, sometimes also referred to as Moldovenesc—local Romanian written with Cyrillic characters), and they speak only incorrect Russian and few words of Ukrainian. This linguistic duality is currently seen as an advantage for both obtaining a Romanian (thus, European Union) passport and importing contraband cigarettes into Romania, which is a profitable job in the area (Cassidy, 2017).

Considering that, before border creation, the two groups of Romanians possessed homogeneous LEK, the different political and, therefore, socioeconomic trajectories experienced by Romanians on the two sides of the border may have shaped the current LEK. On the one hand, we could not find the main drivers of LEK change among Romanians living in Romania as the area was very limitedly touched by the *sistematizarea* policies (rural systematization) implemented by Ceauşescu because of the resistance of local inhabitants to collectivization, the limited interactions of the population with the communist regime, and the unfavorable geographical conditions (Olaru, 2019). On the other hand, after border creation and the consequent annexation of Northern Bukovina to the USSR, Romanians living in Ukraine underwent a process of assimilation into Soviet culture (or Sovietization). Despite the fact that, in the study area, the Romanian language has prevailed for a long time in both schools and churches (considered the most important local authorities), villagers underwent a process of indirect assimilation into Russian culture, also through the adoption of the Cyrillic alphabet to write the Romanian language (Popescu, 1994). With regard to medicinal knowledge, the assimilation process could have also been fostered by the evolution of the Northern Bukovinian health system during and after the collapse of the USSR in 1991. According to the historical analysis proposed by Lekhan et al. (2010), during the Soviet era, the medical system provided universal access to health services, and pharmaceutical products were well distributed at the local level. Despite their wide availability, the quality of such pharmaceuticals were not high as medical guidance was mostly based on “expert” advice rather than on evidence-based medicine (Danichevski et al., 2008; Rechel et al., 2011). Therefore, several medical treatments were ineffective despite the country having one of the highest numbers of physicians per capita (Cromley and Craumer 1990; Rechel et al., 2011). The creation of the independent state of Ukraine and the hard shift from a communist to a market economy resulted in a decline in population health as well as a high cost of medical supplies (Lekhan et al., 2010).

This health context may have promoted the use of local resources, especially in the economic crisis of the 1990s, when many people could read Russian (and the books published in this language), and medicinal products were rare and expensive. As soon as the economic situation improved, Romanians living in Ukraine, in order to make a profit from their ability to navigate multilinguistic and multicultural systems, started emigrating and obtaining medicines from other countries as well as obtaining remittances to be able to buy from local pharmacies.

The introduction of Soviet elements into the local Romanian (and formerly Austro-Hungarian) culture also promoted the ability of local inhabitants to obtain access to Russian books as medicinal sources of knowledge, thus introducing some global (pan-Soviet) elements into the local (Romanian) ethnomedicine. This new local knowledge resulted in the current higher number of plant taxa used for medicinal purposes but in their less consistent use (compared to Romanians living in Romania).

What emerges from this analysis is the inner border of Romanians living in Ukraine, e.g., a cultural border also found in other communities living in the proximity of political borders (e.g., Mattalia et al., 2020a). Indeed, they hold Ukrainian passports, but they mostly share only the Soviet era with Ukrainians as only younger generations can speak some Ukrainian. As they clearly explained to us, they are Romanians who happened to be included in the Soviet Union. Therefore, while sharing with Ukrainians some (pan-) Soviet characteristics, such as the love of books and the consequent tendency toward knowledge standardization, Romanians living in Ukraine also share some Romanian elements as they watch Romanian television and, in a great majority of cases, their parents were born in a Romanian environment. Therefore, paraphrasing Marsico, (2016), they belong to the two sides without being defined by either of the 2 parts. Indeed, Romanians living in Ukraine are an interesting case of “unbelongingness.” This is probably the result of a forced assimilation into the dominant Soviet culture and the consequent loss of some pieces of Romanian (formerly Austro-Hungarian) identity. However, the centripetal forces of the USSR did not allow interstices and expanded homogeneously to most of its territories despite the presence of ethnolinguistic diversity. This resulted in a forced “alphabetization” of the last “Latin” island of Romanians into the Slavic world, which had been developed for centuries in other trajectories. This also occurred through precise strategies of science popularization with a few books regarding medicinal plants published several times (for a possible list, see Mattalia et al., 2020b).

## Conclusions

Overall, the results reveal four main findings.

1. The communities living in Ukraine share more LEK than the ones living in Romania. We can argue that, for about 50 years (1940–1991), Northern Bukovina belonged to a larger political system, the USSR, which uniformly delivered health services, equally affecting Hutsul and Romanian medicinal knowledge corpora by integrating homogeneous pan-Soviet (global) elements, as indicated by several plant uses common among the groups living in Ukraine, into the local corpus of ethnomedicinal knowledge, thus creating a glocal ethnomedicinal corpus of knowledge. In addition, Romanians of Northern Bukovina appear to use a smaller number of medicinal plants due to their movement to other European countries where they (proudly) buy foreign medicinal products.
2. The more divergent LEK of Hutsuls and Romanians living in Romania who have been living relatively independently from one another may be due to the lack of any recent strong centralization force in the valley as Ceauşescu’s policies do not appear to have impacted LEK because of their limited implementation in the Bukovinian area. The similarities among the two Romanian communities could instead be due to common historical roots and language and, therefore, possible common sources of knowledge (e.g., Romanian books and television).
3. From the perspective of divergent trajectories of herbal knowledge, we observed substantial differences in LEK transmission across the border. The main difference concerns the use of written and visual sources, which is quite limited among Romanians and Hutsuls living in Romania (where vertical transmission prevails), but is rather important among the bibliophilic communities living in Ukrainian Bukovina.
4. Finally, we found that, in multilanguage communities (such as that of Romanians living in Ukraine), an analysis of plant names can provide important clues to trace the possible origin of such medicinal uses.

 Further research is needed to more thoroughly explore the link between wild plants and the way people refer to them in order to understand the implemented strategies of LEK transmission in multicultural contexts.

## Data Availability

The datasets generated for this study are available from the authors upon reasonable request. Data from ERC project DiGe will be fully available after the project ends.
